# Effects of *Glomus intraradices* Inoculation on Growth, Nutrient Uptake, and Rhizosphere–Endophytic Microenvironment of Sweet Potato Seedlings

**DOI:** 10.3390/jof12060393

**Published:** 2026-05-29

**Authors:** Jie Yuan, Wenna Zhao, Xiaoqing Wu, Minghui Xu, Cheng Ji, Cong Xu, Fei Chen, Yongchun Zhang, Jidong Wang

**Affiliations:** 1 Institute of Agricultural Resources and Environment, Jiangsu Academy of Agricultural Sciences, Nanjing 210014, China; yuanjie@jaas.ac.cn (J.Y.);; 2 National Agricultural Experimental Station for Agricultural Environment, Luhe, Jiangsu Academy of Agricultural Sciences, Nanjing 210014, China; 3 School of Life Sciences, Nanjing Normal University, Nanjing 210023, China; 4 College of Resources and Environmental Sciences, Nanjing Agricultural University, Nanjing 210095, China

**Keywords:** *Glomus intraradices*, sweet potato, root metabolome, rhizosphere bacteria, endophytic bacteria, predicted functional potential

## Abstract

Arbuscular mycorrhizal fungi (AMF) can improve plant performance, but how they coordinately influence root metabolism and associated bacterial communities in sweet potato remains unclear. Here, a pot experiment was conducted to investigate the effects of *Glomus intraradices* inoculation on sweet potato seedlings by integrating analyses of rhizosphere soil properties, plant growth and nutrient uptake, root metabolomics, and rhizosphere and endophytic bacterial communities using 16S rRNA gene sequencing with FAPROTAX-based functional prediction. AMF inoculation significantly increased whole-plant fresh and dry biomass, potassium concentration and accumulation, and the accumulation of starch and water-soluble carbohydrates, while no significant effects were observed on dry matter rate or plant nitrogen and phosphorus concentration. In the rhizosphere, AMF reduced soil electrical conductivity and increased organic matter content without significantly affecting pH, alkali-hydrolyzable nitrogen, available phosphorus, or available potassium. Root metabolomic profiling identified 289 differential metabolites, with enrichment of phenylpropanoid biosynthesis, glycerophospholipid metabolism, porphyrin metabolism, and nucleotide metabolism, together with broad up-regulation of lipid-related metabolites. Bacterial communities showed strong compartment specificity, with the root endosphere displaying lower alpha diversity than the rhizosphere. Higher rhizosphere bacterial Shannon diversity was observed in the AMF treatment, together with compartment-dependent shifts in bacterial community composition; enrichment of endophytic taxa such as *Devosia* and *Niastella* was detected following AMF inoculation. Functional prediction further suggested niche differentiation between rhizosphere and endophytic bacteria, together with AMF-associated shifts in carbon- and nitrogen-related functions. Overall, these results suggest that *G. intraradices* inoculation is associated with enhanced sweet potato growth and enhanced potassium and carbohydrate accumulation in association with coordinated changes in rhizosphere conditions, root metabolism, and bacterial community assembly.

## 1. Introduction

Arbuscular mycorrhizal fungi (AMF) form symbiotic associations with approximately 80% of terrestrial plants and play important roles in nutrient exchange and plant adaptation to environmental stress [1,2,3,4]. Through colonization of host roots, AMF can enhance the acquisition of water and mineral nutrients, particularly phosphorus (P), nitrogen (N), and potassium (K), in exchange for photosynthetically derived carbon [5,6,7,8,9]. In the context of sustainable agriculture, AMF have received increasing attention as potential biofertilizers and bioprotectants for improving crop productivity and soil quality [10,11,12,13].

Sweet potato (*Ipomoea batatas* (L.) Lam.) is an important food and industrial crop worldwide because of its high yield potential and nutritional value [14]. Potassium is particularly critical for sweet potato production, as it is closely associated with root enlargement, starch accumulation, and stress resistance [14,15,16,17,18]. However, excessive potassium fertilizer input increases production costs and may lead to environmental risks. Therefore, developing biological strategies to improve potassium use efficiency in sweet potato is of considerable practical importance [19,20]. Although AMF have been reported to enhance potassium uptake in several crops [5,6,7,21,22,23], the processes associated with this response remain insufficiently understood, especially in sweet potato.

Previous studies have shown that AMF can modify rhizosphere soil properties and alter bacterial community composition [4,24,25,26]. As a hotspot of plant–microbe interactions, the rhizosphere plays an essential role in nutrient cycling, microbial recruitment, and plant health [10,27,28,29]. Thus, most studies have focused primarily on the rhizosphere, whereas the endophytic bacterial community, which inhabits internal root tissues and interacts more directly with the host plant, has received comparatively less attention [27,30,31,32]. The root endosphere functions as a selective compartment that filters bacteria from the rhizosphere [33,34], but whether AMF influence this filtering process and thereby contribute to the selective enrichment of beneficial endophytes remains unclear [35]. In addition, prediction-based assessments of bacterial functional potential under AMF inoculation, such as those related to nitrogen transformation or organic matter degradation, remain limited.

In parallel, metabolomics studies have shown that AMF colonization is often accompanied by substantial metabolic reprogramming in host roots, including changes in phenylpropanoid metabolism, lipid metabolism, and phytohormone-related pathways [10,21,36,37]. Among these, the phenylpropanoid pathway is of particular interest because it contributes to the biosynthesis of lignin, flavonoids, and antimicrobial compounds involved in plant defense and microbe-mediated signaling [7,36,38,39]. Nevertheless, the relationships between AMF-associated metabolic shifts and changes in bacterial community assembly, particularly the selective enrichment of specific endophytic taxa, remain poorly understood in sweet potato.

To address these gaps, we conducted a pot experiment under controlled conditions to evaluate the responses of sweet potato seedlings to AMF inoculation. We hypothesized that AMF inoculation would increase sweet potato biomass and nutrient and carbohydrate accumulation, alter root metabolic profiles, and be associated with shifts in bacterial community composition and predicted functions in both the rhizosphere and root endosphere. Specifically, we aimed to: (1) assess the effects of AMF on plant biomass, nutrient uptake, and carbohydrate accumulation; (2) characterize AMF-associated changes in root metabolic profiles and identify major responsive pathways and metabolites; (3) examine the responses of rhizosphere and endophytic bacterial communities, including diversity, composition, and predicted functional potential, to AMF inoculation; and (4) integrate these datasets to develop an overall framework for understanding how AMF-associated changes in the rhizosphere environment, root metabolism, and bacterial community assembly may contribute to sweet potato growth and nutrient accumulation.

## 2. Materials and Methods

### 2.1. Experimental Materials

This study employed sandy loam fluvo-aquic soil (Eutric Cambisol) collected from Jiangyan City in Jiangsu Province [21,22]. With a pH of 7.13, the soil contained 66 mg kg^−1^ available potassium (AK), 49 mg kg^−1^ available nitrogen (alkali-hydrolyzable N, AN) and 23 mg kg^−1^ available phosphorus (Olsen-P, AP), indicating moderate nitrogen and phosphorus availability but comparatively low potassium status.

Sweet potato Su 16, bred by the Jiangsu Academy of Agricultural Sciences, was selected for its universality of cultivation. *Glomus intraradices* BEG141 was selected based on previous studies [40]. AMF inoculum containing *G. intraradices* spores (about 120 spores g^−1^) was propagated using *Trifolium repens* [21].

### 2.2. Experimental Design

This study was carried out in a greenhouse at the Jiangsu Academy of Agricultural Sciences in Nanjing, China, during May and June of 2024, with the experimental cultivation lasting for one month. A pot experiment was conducted with two treatments, *Glomus intraradices* inoculation (AMF group) and non-inoculation (CK group), with 6 replicates per treatment. Each pot was filled with 7 kg of treated soil. In the AMF group, 25 g of *G. intraradices* inoculant was added to each pot and evenly mixed with the soil. In the CK group, an equal amount of sterilized inoculant matrix (sterilized at 121 °C for 20 min) was added to exclude the influence of the matrix itself on the experiment [22]. Seedlings with consistent growth were selected, and 1 seedling was transplanted per pot. The soil water content was controlled within 60–70% of field capacity to ensure appropriate moisture conditions. Samples were harvested after 30 days of cultivation (sweet potato seedling stage) for the determination of various indexes [21].

### 2.3. Plant Sampling and Analysis

At the seedling stage, specifically 30 days after inoculation, six pots were collected from each treatment for harvest and subsequent analysis. Fresh roots of three pots from each treatment were used for mycorrhizal colonization analysis and endophytic bacterial community analysis. The intensity of mycorrhizal colonization in roots was evaluated microscopically at 40× magnification using an SMZ745T microscope (Nikon, Tokyo, Japan) [21,41]. The infection rate was about 48%. Fresh roots were taken, rinsed with deionized water, surface-disinfected by immersion in 75% ethanol for 30 s, followed by treatment with 3% sodium hypochlorite for 3 min and three rinses with sterile water, then rapidly frozen in liquid nitrogen and preserved at −80 °C for subsequent analysis of the endophytic bacterial community [42,43]. For each treatment, three additional pots were harvested and separated into shoots and roots. The plant materials were initially dried at 105 °C for 30 min and then maintained at 75 °C until constant mass, after which dry matter was recorded. The dried samples were subsequently ground, passed through a 0.25 mm sieve, and subjected to homogenization. Afterwards, 0.5 g portions were digested with a mixed acid system of H_2_SO_4_ and H_2_O_2_. Following digestion, nitrogen content was measured by the Kjeldahl method [44], phosphorus concentration was quantified using the molybdenum blue method [45], and potassium concentration was determined by flame photometry [19]. The water-soluble carbohydrates (WSCs) and starch concentration were determined by the colorimetric phenol–sulfuric acid method [46]. The accumulation of plant nutrients or carbohydrates was calculated by multiplying dry matter by the respective concentrations of each component.

### 2.4. Root Untargeted Metabolome Analysis

Untargeted metabolomic profiling of root samples was conducted by ultra-high-performance liquid chromatography coupled with tandem mass spectrometry (LC-MS/MS; Vanquish, Thermo Fisher Scientific, Waltham, MA, USA) [21]. Metabolites were separated on an Acquity UPLC HSS T3 column (2.1 × 100 mm, 1.8 μm, Waters, Santa Clara, CA, USA) using a binary mobile phase consisting of solvent A, an aqueous mixture containing 5 mM acetic acid and 5 mM ammonium acetate, and solvent B, acetonitrile. The injection volume was set at 2 μL, and the sample tray was maintained at 4 °C. High-resolution spectra were acquired on an Orbitrap Exploris 120 platform in both full-scan MS and MS/MS acquisition modes. To monitor instrumental performance and analytical reproducibility, pooled quality-control samples were generated by mixing equal volumes of supernatants from all individual samples. After data acquisition, the raw files underwent format conversion, peak detection, and signal integration, and metabolite annotation was performed using the HMDB and KEGG COMPOUND databases.

Metabolite data were analyzed using partial least squares discriminant analysis (PLS-DA) [21]. Compounds with a variable importance in projection (VIP) value greater than 1 were first identified as candidate differentially abundant metabolites. These candidates were then screened further by integrating Student’s t-test outcomes, univariate *p*-values, and fold-change (FC) metrics to determine statistically significant differences in abundance. Differential expression is defined as log_2_*FC* > 0 (up-regulated) or log_2_*FC* < 0 (down-regulated). Then, KEGG topology analysis was conducted on AMF up-regulated and down-regulated metabolites.

### 2.5. Rhizosphere and Bulk Soil Physicochemical Property Analysis

Root systems were gently excavated together with the surrounding rhizosphere soil, after which 1–2 mm of rhizosphere soil adhering to roots was subsequently harvested [21]. Following the removal of large particles by passing the soil through a 2 mm sieve, each sample was divided into two subsamples: one portion was air-dried for the determination of soil chemical properties, whereas the other was immediately stored at −80 °C for subsequent DNA extraction.

Soil pH was assessed with a glass-electrode pH meter in a soil–water suspension prepared at a ratio of 1:5 [47]. Electrical conductivity (EC) was then analyzed from the same suspension using a conductivity meter [19]. Soil organic matter (SOM) content was quantified by the potassium dichromate oxidation procedure [48]. Available nitrogen was evaluated through the alkaline hydrolysis diffusion method, followed by separation and acid-base titration with a polypropylene diffusion dish [21]. Available phosphorus was extracted with NaHCO_3_ and subsequently determined by the molybdenum–antimony colorimetric method [49]. Available potassium was extracted using NH_4_OAc and measured by flame photometry [19].

### 2.6. Rhizosphere and Endophytic Bacterial Community Composition and Function Analysis

Total genomic DNA was isolated from the samples with the Power Soil^TM^ Kit (QIAGEN Laboratories, Carlsbad, CA, USA) according to the supplier’s protocol [21]. DNA integrity and purity were evaluated spectrophotometrically, and nucleic acid concentration was further quantified using a NanoDrop ND-2000 instrument (NanoDrop, Wilmington, DE, USA). The purified DNA was subsequently preserved at −20 °C prior to molecular analysis. To characterize bacterial community composition and diversity, the V3–V4 hypervariable region of the 16S rRNA gene was amplified with primers 338F and 806R [21], and the amplicons were sequenced on the Illumina NextSeq 2000 platform [50]. The resulting raw sequences were merged, denoised, and quality-filtered to generate reliable high-quality reads for subsequent analyses. Based on these processed data, operational taxonomic units (OTUs) were clustered and taxonomically annotated through comparison with the SILVA bacterial reference database.

For each sample, the structural characteristics of the bacterial community were evaluated across multiple taxonomic ranks according to OTU abundance and annotation data. Differences in microbial community composition among treatments were further explored by principal component analysis (PCA) based on Bray–Curtis distances using the “vegan” package in R 4.3.3. In addition, permutational multivariate analysis of variance (PERMANOVA) with 999 permutations, implemented through the Adonis method, was applied to determine whether community composition differed significantly among treatments. To further detect taxa showing distinct enrichment under different fertilization regimes, linear discriminant analysis effect size (LEfSe) was conducted with an LDA threshold greater than 2.5.

Subsequently, bacterial metabolic functions were predicted using the FAPROTAX database and classified into 56 groups, and the functional differences among samples were evaluated through non-metric multidimensional scaling (NMDS) using Bray–Curtis dissimilarity as the distance metric [51]. Additionally, bacterial-associated carbon and nitrogen cycling functions were analyzed and also showed significant differences among treatments. Among all functions, the relative abundances of 28 functions differed significantly among treatments (Table S6).

### 2.7. Statistical Analysis

Differences between two treatment groups were evaluated by an independent-samples *t*-test, and statistical significance was denoted by asterisks (* *p* < 0.05, ** *p* < 0.01, *** *p* < 0.001) [21]. When more than two treatments were compared, one-way analysis of variance (ANOVA) was applied, followed by Tukey’s post hoc multiple-comparison test at the *p* < 0.05 level; significant differences among groups were identified using different lowercase letters. Mycorrhizal growth response (MGR) was calculated [26]. MGR (%) = (value_AMF_ − value_CK_)/value_CK_ × 100%, where “value_AMF_” was the value in a mycorrhizal pot, and “value_CK_” was the mean value of the corresponding control treatment.

## 3. Results

### 3.1. Effects of AMF on Physicochemical Properties of Rhizosphere Soils

**Figure 1:**
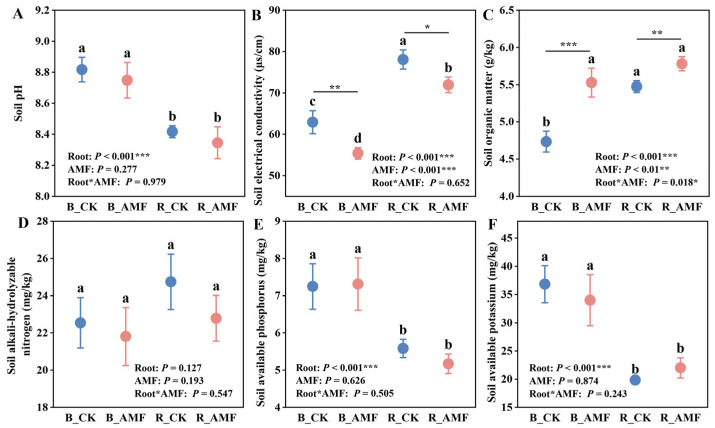
Effects of AMF on physicochemical properties of bulk and rhizosphere soils. (**A**) Soil pH, (**B**) electrical conductivity, (**C**) organic matter, (**D**) alkali-hydrolyzable nitrogen, (**E**) available phosphorus, and (**F**) available potassium. B_CK, bulk soil from the non-AMF treatment; B_AMF, bulk soil from the AMF treatment; R_CK, rhizosphere soil from the non-AMF treatment; R_AMF, rhizosphere soil from the AMF treatment. Distinct lowercase letters indicate statistically significant differences among the treatment groups at *p* < 0.05. In addition, asterisks represent different significance levels, with * corresponding to *p* < 0.05, ** to *p* < 0.01, and *** to *p* < 0.001.

AMF inoculation significantly reduced electrical conductivity and increased organic matter concentration in rhizosphere soils, with no significant effects on pH, alkali-hydrolyzable nitrogen, available phosphorus, or available potassium (Figure 1). These results indicate that AMF likely promote sweet potato growth through mechanisms other than activating soil nutrients, which is consistent with our findings from another AMF strain [21]. Moreover, the increase in organic matter content further confirms that AMF enhance soil carbon sequestration, possibly due to the secretion of glomalin and other organic substances [23,52].

### 3.2. Effects of AMF on Sweet Potato Biomass, Nutrient Uptake, and Carbohydrate Accumulation

**Figure 2:**
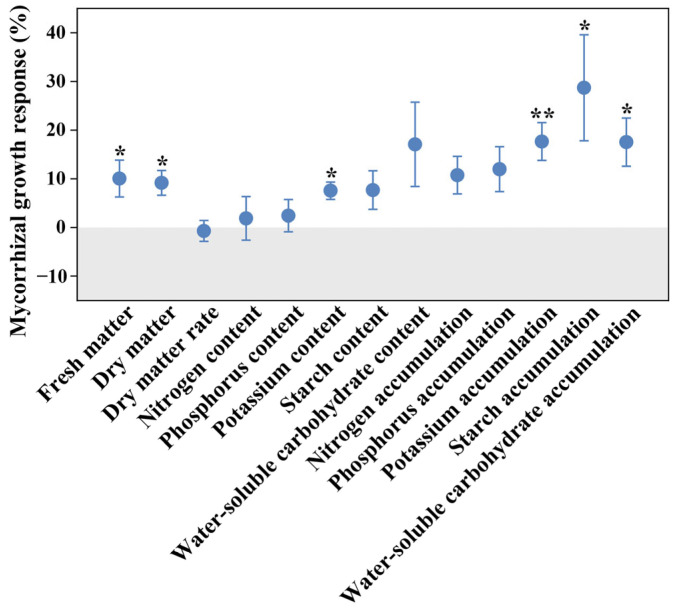
Mycorrhizal growth response of sweet potato biomass, nutrient uptake, and quality traits. Asterisk symbols denote significance thresholds at * *p* < 0.05 and ** *p* < 0.01 probability levels.

AMF significantly improved sweet potato biomass by increasing both fresh and dry weights, although the dry matter rate remained unchanged (Figure 2). For nutrient uptake, AMF selectively increased whole-plant potassium concentration and accumulation, while exerting no significant effect on nitrogen or phosphorus uptake. In addition, AMF significantly promoted the accumulation of starch and water-soluble carbohydrates, without altering their respective concentrations. Thus, AMF enhanced sweet potato biomass and specifically promoted potassium and carbohydrate accumulation, without affecting nitrogen, phosphorus, or constituent concentrations.

### 3.3. Effects of AMF on Root Metabolite Profiles and Metabolic Pathway

**Figure 3:**
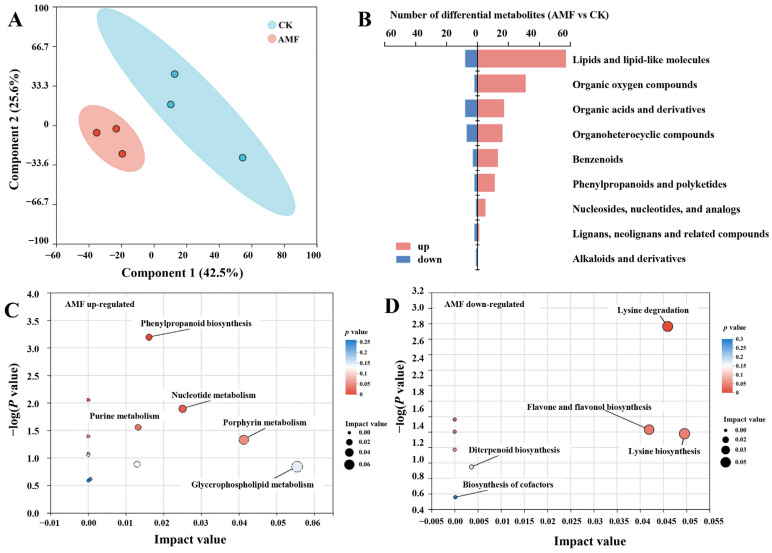
(**A**) Partial least squares-discriminant analysis (PLS-DA) of differentially expressed metabolites (DEMs) in sweet potato root after AMF inoculation. (**B**) Number of differentially expressed metabolites for AMF vs. CK. KEGG topology analysis of AMF up-regulated (**C**) and down-regulated metabolites (**D**). Each bubble represents a metabolic pathway; its color indicates the significance level (−log(*p* value)), ranging from blue (higher) to red (lower), and its size reflects the pathway impact score from the topology analysis.

Among 3848 identified root metabolites, AMF significantly altered 289 (7.51%), with 241 up-regulated and 48 down-regulated (Figure S1). PLS-DA demonstrated a clear separation in metabolite profiles between the AMF-treated group and the CK control group (Figure 3A). Up-regulated metabolites were predominantly lipids and lipid-like molecules (27.0%), phenylpropanoids and polyketides (16.7%), organic acids and derivatives (14.4%), organic oxygen compounds (13.1%), and organoheterocyclic compounds (11.2%) (Figure 3B). In contrast, down-regulated metabolites mainly comprised lignans, neolignans, alkaloids, and their derivatives (Figure 3B). KEGG topology analysis showed that AMF up-regulated pathways involved in glycerophospholipid metabolism, porphyrin metabolism, nucleotide metabolism, and phenylpropanoid biosynthesis, while down-regulating lysine biosynthesis/degradation and flavone/flavonol biosynthesis (Figure 3C,D). The phenylpropanoid biosynthesis pathway showed the most significant up-regulation (*p* = 0.002), whereas lysine degradation was the most down-regulated (*p* = 0.002).

**Figure 4:**
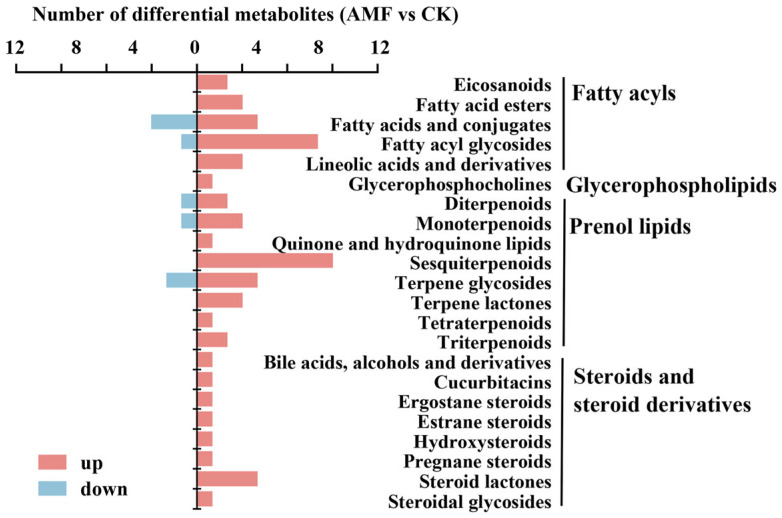
AMF differentially regulated metabolites associated with lipids and lipid-like molecules. Red and blue bars represent numbers of AMF up- and down-regulated metabolites.

For lipids and lipid-like molecules, AMF broadly up-regulated fatty acid derivatives, glycerophosphocholine, multiple terpenoids (di-, mono-, sesqui-, tri-, tetra-terpenoids; terpene glycosides; terpene lactones), quinone/hydroquinone lipids, and various steroids (Figure 4). For phenylpropanoids and polyketides, several metabolites including rutarin, herbimycin A, lymecycline, hydroxysyringaresinol, kuwanol C, epothilone C, epicatechin derivatives, and zearalenone, as well as ansamitocin P-3 and retaspimycin, were up-regulated by AMF (Figure S2). Notably, most phytohormones remained unchanged, whereas gibberellin A38 glucosyl ester, 2-Cis,4-Trans-Xanthoxin, and (+)-dehydrovomifoliol were up-regulated (Table S1).

### 3.4. Effects of AMF on Rhizosphere and Endophytic Bacterial Diversity and Composition

**Figure 5:**
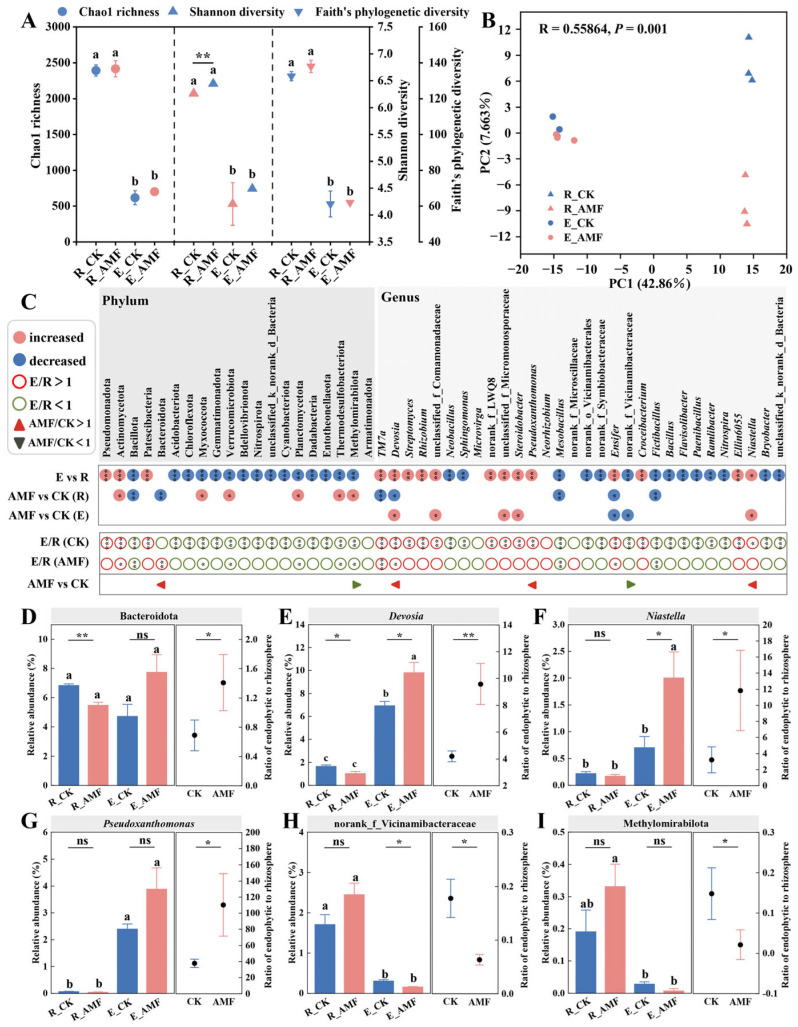
(**A**) Alpha diversity, (**B**) PCA, and (**C**) composition of bacterial community regulated by root and AMF. Six key taxa include Bacillota (**D**), *Devosia* (**E**), *Niastella* (**F**), *Pseudoxanthomonas* (**G**), norank_o_Vicinamibacterales (**H**), and Methylomirabilota (**I**) regulated by root and AMF. E, endophytic bacterial community; R, rhizosphere bacterial community; E/R, ratio of endophytic to rhizosphere. Distinct lowercase letters indicate statistically significant differences among treatment groups at *p* < 0.05. In addition, asterisks represent different significance levels, with * corresponding to *p* < 0.05, ** to *p* < 0.01, and *** to *p* < 0.001. “ns” indicates no significant difference.

Compared with rhizosphere bacteria, endophytic bacteria showed markedly reduced alpha diversity (Chao1, Shannon, and Faith’s PD indexes) and a distinct community composition (Figure 5A,B). AMF significantly increased bacterial Shannon diversity and reshaped the community composition of the rhizosphere bacterial community, rather than that of the endophytic bacterial community (Figure 5A,B). Rhizosphere bacteria mainly belong to Pseudomonadota, Bacillota, and Actinomycetota, whereas endophytic bacteria mainly consist of Pseudomonadota, Patescibacteria, and Actinomycetota (Figure S3A). At the genus level, endophytic bacteria mainly consist of TM7a, *Devosia*, *Streptomyces*, and *Rhizobium* (Figure S3B). Compared with rhizosphere bacteria, three phyla, including Pseudomonadota, Actinomycetota, and Patescibacteria, and 13 genera including TM7a, *Devosia*, *Streptomyces*, *Rhizobium*, *Steroidobacter*, *Pseudoxanthomonas*, *Ensifer*, *Croceibacterium*, *Ellin6055*, and *Niastella* were significantly enriched in endophytic bacteria (Figure 5C; Table S2). LefSe analysis also identified distinct microbial biomarkers across treatments, with 36 genera enriched in the root endosphere versus 136 in rhizosphere soil (Table S3). This root endosphere was marked with the genera containing *TM7a*, *Devosia*, *Streptomyces*, *Rhizobium*, *Pseudoxanthomonas*, *Ensifer*, *Niastella*, etc. (Table S3).

In the root endosphere, AMF-CK contrasts revealed weaker effects in (20 vs. 5 enriched genera) compared to rhizosphere soil (32 vs. 16) (Tables S4 and S5). Rhizosphere bacteria of the AMF group were marked with *Nitrosomonas*, *Aeromicrobium*, *Sandaracinus*, etc. (Table S4), while endophytic bacteria of the AMF group were marked with *Niastella*, *Noviherbaspirillum*, *Ohtaekwangia*, *Acidibacter*, etc. (Table S5). Moreover, AMF significantly enriched Actinomycetota, Myxococcota, Verrucomicrobiota, Planctomycetota, Thermodesulfobacteriota, and Methylomirabilota, and decreased the relative abundance of Bacillota, Bacteroidota, *TM7a*, *Devosia*, *Mesobacillus*, *Ensifer*, and *Fictibacillus* in rhizosphere soil; however, they enriched *Devosia*, unclassified_f_Comamonadaceae, unclassified_f_Micromonosporaceae, *Steroidobacter*, and *Niastella*, and reduced *Ensifer* and norank_f_Vicinamibacteraceae in the endophytic bacterial community (Figure 5C; Table S2). We further analyzed the selective enrichment of endophytic bacteria from the rhizosphere in the CK and AMF treatment (Figure 5C; Table S5). Compared with CK, AMF enhanced the enrichment of Bacteroidota, *Devosia*, *Niastella*, and *Pseudoxanthomonas* from the rhizosphere into the root, while limiting the enrichment of norank_f_Vicinamibacteraceae and Methylomirabilota (Figure 5D–I; Table S6). These results indicate that the endophytic bacterial community is distinctly structured from the rhizosphere community, with AMF playing a selective role in enriching specific genera such as *Devosia* and *Niastella*, while suppressing others like norank_f_Vicinamibacteraceae.

### 3.5. Effects of AMF on Rhizosphere and Endophytic Bacterial Functions

**Figure 6:**
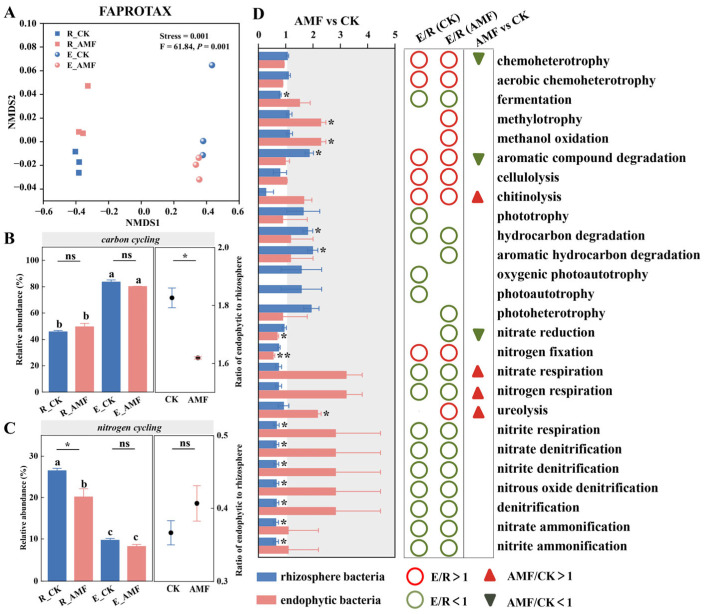
(**A**) NMDS of bacterial functions for different groups. (**B**) Carbon cycling, (**C**) nitrogen cycling, and (**D**) 28 functions regulated by root and AMF. E/R, ratio of endophytic to rhizosphere. Distinct lowercase letters indicate statistically significant differences among treatment groups at *p* < 0.05. In addition, asterisks represent different significance levels, with * corresponding to *p* < 0.05, and ** to *p* < 0.01. “ns” indicates no significant difference.

The functional profiles of rhizosphere and endophytic bacterial communities were predicted using the FAPROTAX database, and carbon and nitrogen cycling were also analyzed. Significant functional differentiation was observed between the two niches (Figure 6A), with endophytic bacteria enriched in carbon cycling, chemoheterotrophy, aerobic chemoheterotrophy, aromatic compound degradation, cellulolysis, chitinolysis, and nitrogen fixation, while rhizosphere bacteria were significantly dominant in 18 functional categories including nitrogen cycling, fermentation, phototrophy, hydrocarbon degradation, and aromatic hydrocarbon degradation (Table S7; Figure 6B,C).

AMF further reshaped the functional profiles of both rhizosphere and endophytic bacterial communities (Figure 6A). In the rhizosphere soil, AMF significantly enhanced the functions of aromatic compound degradation, hydrocarbon degradation, and aromatic hydrocarbon degradation, while significantly reducing nitrogen cycling, and fermentation (Figure 6B–D). In the endophytic bacteria, AMF inoculation significantly increased methylotrophy, methanol oxidation, and ureolysis, but significantly decreased nitrate reduction and nitrogen fixation (Figure 6B–D). Additionally, AMF increased the enrichment of bacterial functions associated with chitinolysis, nitrate reduction, nitrate respiration, nitrogen respiration, and ureolysis, while limiting the functions related to carbon cycling, chemoheterotrophy, aromatic compound degradation, and nitrate reduction (Figure 6B,D). Thus, rhizosphere and endophytic bacteria naturally have a clear functional division, with endophytic bacteria involved in carbon cycling and nitrogen fixation, while rhizosphere bacteria are involved in nitrogen cycling and fermentation. Moreover, AMF help plants utilize nitrogen more efficiently by finely regulating the carbon and nitrogen metabolism of bacterial communities.

### 3.6. Integrative Analysis and Conceptual Model

**Figure 7:**
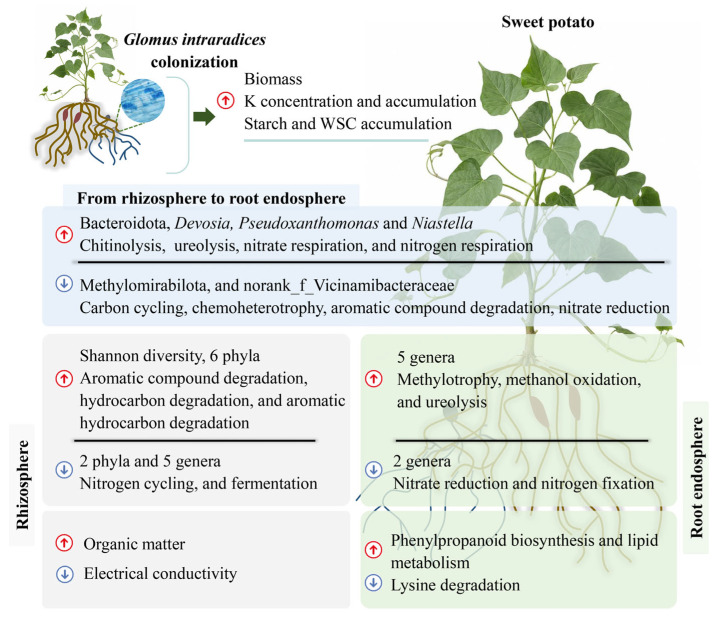
Conceptual model of AMF *Glomus intraradices* colonization regulating plant performance and root metabolism and enriching endophytic bacteria from rhizosphere soil of sweet potato seedlings. A red circle-enclosed upward arrow signifies a rise in the indicator; a blue circle-enclosed downward arrow signifies a fall.

We propose a conceptual model of AMF colonization in which it regulated plant performance and root metabolism and enriched endophytic bacteria from the rhizosphere soil of sweet potato seedlings (Figure 7). AMF altered the rhizosphere environment (increased organic matter, decreased electrical conductivity), up-regulated root metabolism (phenylpropanoid biosynthesis and lipid metabolism), reshaped bacterial community composition (especially by selectively enriching *Devosia* and *Niastella* from the rhizosphere to the endosphere), and redirected predicted bacterial functions (toward chitinolysis and ureolysis). These changes converged on improved plant performance: enhanced potassium and carbohydrate accumulation, and increased biomass without altering dry matter rate. This study provides a systematic description of AMF-induced changes in sweet potato. However, causal links remain to be validated using larger sample sizes or genetic approaches.

## 4. Discussion

### 4.1. AMF Promoted Sweet Potato Growth, Potassium Uptake, and Carbohydrate Accumulation

Our results show that AMF significantly increased whole-plant biomass, potassium uptake, and total starch and water-soluble carbohydrate accumulation, but did not significantly affect dry matter rate, nitrogen or phosphorus, or carbohydrate concentrations (Figure 2). Soil analysis revealed that AMF reduced electrical conductivity and increased organic matter, with no changes in pH, alkali-hydrolyzable N, available P, or available potassium (Figure 1). These results suggest that AMF likely promoted sweet potato growth through mechanisms other than increasing soil nutrient availability, which is consistent with our previous findings obtained with another AMF strain [21]. In addition to plant responses, AMF inoculation altered several soil properties. The decrease in electrical conductivity may be associated with improved soil structure and reduced soluble salt accumulation linked to AMF hyphal activity, whereas the increase in organic matter may be partly associated with glomalin-related soil protein (GRSP) and other extracellular polymeric substances [23,52,53]. GRSP has been reported to be a relatively recalcitrant carbon-containing soil component and may contribute to soil organic carbon stabilization [54,55]. Such changes may contribute to a more favorable rhizosphere environment for plant growth and microbial activity. Moreover, previous studies have increasingly recognized that AMF-associated carbon inputs may have broader implications for soil health and long-term soil carbon dynamics [52,56]. Together, these soil responses suggest that although AMF modified the belowground environment, the growth-promoting effect observed here is unlikely to be explained solely by enhanced soil nutrient availability.

Notably, the concentrations of starch and water-soluble carbohydrates remained unchanged despite their increased accumulation (Figure 2), indicating that greater biomass production was accompanied by proportional carbohydrate accumulation. This pattern suggests that AMF may enhance whole-plant carbon assimilation, thereby increasing total carbohydrate accumulation without altering carbohydrate concentrations. This interpretation is broadly consistent with previous reports showing that AMF symbiosis can alter host carbon partitioning and metabolic priorities in other crops. In tomato, *R. irregularis* and *F. mosseae* symbiosis increased sugar metabolic intermediates while reducing certain amino acids [57]. In peanut, once symbiosis with *F. mosseae* became fully established, overall metabolic activity shifted markedly toward the biosynthesis of lipids, amino acids, carboxylic acids, and carbohydrates; notably, the proportion of lipid-related compounds rose by 8.5% compared with the pre-symbiotic stage [36]. In our previous study, *Claroideoglomus etunicatum* reprogrammed sweet potato root metabolism and was associated with metabolic patterns consistent with enhanced stress adaptation and nutrient uptake, together with broader changes in root hormone-related pathways under low-potassium conditions, including the up-regulation of gibberellin A70, abscisic acid, indole-3-acetic acid, and cis-zeatin [21]. In this study, the up-regulation of gibberellin A38 glucosyl ester and dehydrovomifoliol (Table S1) suggests a possible involvement of gibberellin- and abscisic acid-related signaling in AMF-associated physiological regulation.

### 4.2. AMF Reprogrammed Root Metabolism, Particularly Phenylpropanoid and Lipid-Related Pathways

AMF colonization extensively reprogrammed sweet potato root metabolism, most notably by up-regulating the phenylpropanoid biosynthesis pathway and a broad range of lipid-related metabolites, while down-regulating flavone/flavonol biosynthesis and lysine degradation pathways (Figure 3 and Figure S2). In *Lycium barbarum*, AMF inoculation has been reported to increase the activities of key enzymes in phenylpropane metabolism and boost lignin and flavonoid production, leading to enhanced disease resistance [58]. We identified several up-regulated compounds related to phenylpropanoid and polyketide metabolism (Figure S2), including rutarin, epicatechin-related metabolites, and herbimycin A, many of which have been reported to possess antimicrobial activity [59,60]. Their accumulation may be associated with shifts in root chemical environments and potentially related signaling processes during AMF colonization. Because flavonoid-related pathways are closely linked to phenylpropanoid metabolism, the down-regulation of flavone and flavonol biosynthesis is notable in the context of simultaneous induction of other phenylpropanoid-related compounds. Flavonoids are widely recognized as signaling compounds in plant–microbe interactions; however, their roles in AMF symbiosis are complex and highly context-dependent. Different flavone and flavonol compounds may either promote or inhibit AMF colonization, depending on compound identity and biological context [61]. Once AMF colonization had been established in the roots, suppression of this pathway may be associated with a reorientation of root signaling and carbon allocation after colonization. Rather than continuing to invest heavily in pre-colonization signaling, the observed metabolic pattern may reflect a relative shift in resource allocation toward maintenance of the established mycorrhizal association. This shift in metabolic allocation may also help explain why total carbohydrate accumulation increased while carbohydrate concentrations remained unchanged (Figure 2).

Beyond changes in signaling-related secondary metabolism, AMF colonization was also accompanied by pronounced remodeling of root lipid metabolism. Lipids accounted for 27.0% of all up-regulated metabolites, covering fatty acid derivatives, glycerophosphocholine, terpenoids, terpene glycosides, steroids, and related compounds (Figure 4). AMF colonization is well known to alter host lipid and fatty acid metabolism, supporting fungal growth and symbiotic functioning [62,63]. This lipid remodeling is not limited to carbon supply; it also has important structural implications for symbiotic interface formation. Changes in glycerophospholipid composition are important for arbuscule accommodation and for keeping the perifungal membrane interface functional [64]. Terpenoids and steroids, meanwhile, are often linked with membrane stability and protection against stress [65,66]. Similar results have been reported in maize, where metabolomic analyses showed that AMF inoculation affected lipid, fatty acid, terpenoid, and phenylpropanoid metabolism simultaneously [67]. In tomato, mycorrhizal symbiosis induces a broad metabolic transition from amino acid accumulation toward oxylipins and products derived from α-linolenic acid metabolism [57]. Collectively, this broad reprogramming of lipid metabolism may help maintain an efficient symbiotic interface, potentially supporting nutrient exchange in AMF-colonized plants. Moreover, the down-regulation of lysine degradation (Figure 3D) is consistent with reports that AMF colonization alters amino acid metabolism in mycorrhizal roots [57], and may also accord with our finding that AMF did not significantly change whole-plant nitrogen content (Figure 2). Taken together, these results indicate that AMF colonization induced a coordinated metabolic transition in sweet potato roots, particularly involving phenylpropanoid- and lipid-related pathways, which may have contributed to a cellular environment favorable for symbiotic functioning and to the observed increases in potassium uptake and carbohydrate accumulation.

### 4.3. AMF Reshape Rhizosphere and Endophytic Bacterial Communities with Compartment-Specific Patterns

The root endosphere serves as a highly selective microbial habitat. Consistent with the well-established root filtering effect [68], we observed significantly lower alpha diversity in the endophytic bacterial community than in the rhizosphere, together with a pronounced separation in community composition between the two compartments (Figure 5A,B). This spatial partitioning was also associated with distinct predicted functional profiles. Endophytic bacteria were more strongly associated with predicted functions related to carbon cycling and nitrogen fixation, whereas rhizosphere bacteria were more associated with predicted nitrogen cycling and fermentation-related functions (Figure 6; Table S6). In the endophytic compartment, the dominant phyla were Pseudomonadota, Patescibacteria, and Actinomycetota. At the genus level, *TM7a*, *Devosia*, *Streptomyces*, *Rhizobium*, *Steroidobacter*, *Pseudoxanthomonas*, and *Niastella* were especially abundant (Figure S3). Taken together, these taxa appear to represent a recurring bacterial assemblage associated with the sweet potato root interior, although this pattern may vary with host genotype or environmental conditions.

AMF colonization further modified these bacterial communities in a compartment-dependent manner. Previous work has shown that AMF inoculation can increase the diversity of rare taxa, thereby stabilizing rhizosphere co-occurrence networks while enriching specific bacterial groups within the endosphere [35]. Consistent with this pattern, we found that AMF colonization significantly enriched Actinomycetota, Myxococcota, Verrucomicrobiota, Planctomycetota, Thermodesulfobacteriota, and Methylomirabilota in the rhizosphere, while increasing the relative abundance of *Devosia*, unclassified_f_Comamonadaceae, unclassified_f_Micromonosporaceae, *Steroidobacter*, and *Niastella* in the root endosphere (Figure 5C; Table S2). These results indicate that AMF were associated not only with shifts in the external rhizosphere microbiota but also with changes in bacterial composition within root tissues.

A recent review of bacteria associated with AMF emphasized that these fungi harbor distinct microbial communities both within and beyond plant roots [69]. These associated bacteria exhibit diverse functional capabilities, including nitrogen fixation, phosphate mobilization, phytohormone production, and biocontrol against pathogens. Likewise, recent research on AMF spores in chernozem soil revealed that AMF spore-associated endophytic bacteria possess broad-spectrum antimicrobial activity (23% of strains) and 1-aminocyclopropane-1-carboxylate deaminase activity (29% of strains), and AMF–bacteria interactions can enhance host stress resistance through mechanisms including phosphorus solubilization, siderophore production, and phytohormone regulation [70]. Against this background, the bacterial shifts observed here may be ecologically relevant to the assembly and functional potential of the AMF-associated root microbiome. Based on functional prediction, AMF inoculation increased the predicted relative abundance of rhizosphere functions associated with aromatic compound and hydrocarbon degradation, while reducing predicted functions related to nitrogen cycling, including denitrification, ammonification, and nitrite respiration (Figure 6). The latter observation aligns with reports that AMF modify nitrogen cycling microbial communities, particularly suppressing denitrification and nitrous oxide emissions [27]. In the endosphere, AMF increased the predicted relative abundance of functions related to methylotrophy, methanol oxidation, and ureolysis, while decreasing those associated with nitrate reduction and nitrogen fixation (Figure 6B–D). This shift suggests that AMF may influence the assembly of an endophytic community with a different inferred functional profile. Relative to the control, this profile appeared less associated with inorganic nitrogen transformation and more associated with organic nitrogen recycling, although these predicted functions were not directly validated. This pattern may also accord with the unchanged plant nitrogen content observed in our study (Figure 2), although direct functional validation is still needed. Thus, AMF may restructure the root microbiome through a combination of rhizosphere reshaping and endosphere filtering, accompanied by shifts in inferred nitrogen-related functional potential across compartments.

To further link community shifts to potential recruitment patterns, we performed enrichment analysis from the rhizosphere to the endosphere (Figure 5E,F). AMF colonization facilitated the selective enrichment or recruitment of Bacteroidota, *Devosia*, *Pseudoxanthomonas*, and *Niastella* into the endosphere, while restricting the endosphere enrichment of Methylomirabilota and norank_f_Vicinamibacteraceae. Notably, several of the enriched endophytic genera have been reported to possess plant-beneficial traits. Devosia is recognized as a common root endophytic genus in multiple plant species [33], and previous studies have shown that it can physically associate with AMF hyphae and may be linked to plant growth and nitrogen uptake [30]. *Pseudoxanthomonas*, frequently isolated as an endophyte, has been reported to improve host salt tolerance in some plant systems [71]. *Niastella* exhibits resistance to heavy metals and encodes enzymatic machinery for chitin degradation and the catabolism of aromatic pollutants [72,73], and its enrichment on maize root surfaces further underscores its affinity for the rhizoplane–endosphere interface [74]. Based on functional inference, AMF appeared to be associated with an endophytic assemblage relatively enriched in predicted functions related to chitinolysis, ureolysis, and nitrate respiration, while reducing those associated with carbon cycling, chemoheterotrophy, aromatic compound degradation, and nitrate reduction (Figure 6), suggesting that AMF may contribute to the selective enrichment of endophytes with particular inferred metabolic potentials, especially those related to organic matter turnover and nitrogen recycling. Collectively, these results suggest that AMF colonization is associated with the enrichment of endophytes with potentially beneficial traits, although their specific contributions to plant growth promotion and stress adaptation within the mycorrhizosphere remain to be verified.

### 4.4. Limitations and Future Perspectives

Several limitations of this study should be acknowledged. First, the direct contribution of AMF hyphae to potassium uptake was not quantified; compartmentalized culture systems combined with isotope tracers such as ^42^K or ^86^Rb are needed to distinguish direct hyphal transport from AMF-induced changes in host root physiology. Second, the relationships between specific root metabolites (e.g., phenylpropanoids) and the selective enrichment of endophytic genera such as *Devosia* and *Niastella* remain correlative, and synthetic community experiments or gnotobiotic systems are required to establish causality. Third, bacterial functional profiles were inferred from amplicon-based taxonomic data rather than validated by metagenomic or biochemical approaches; therefore, these functional shifts should be interpreted as predicted tendencies rather than confirmed activities. Nevertheless, accumulating evidence indicates that AMF can select and recruit beneficial bacterial symbionts through physical, chemical, and biological connections [69]. For example, *F. mosseae* can attract *P. putida* KT2440 by secreting cysteine as a signaling molecule, thereby promoting its colonization in the soybean rhizosphere [24]. Similarly, our recent study showed that *C. etunicatum* may enrich Planctomycetota in sweet potato through root exudates such as indole-2-carboxylic acid, L-arabitol, and tropic acid [21]. Direct metabolomic comparison between *G. intraradices* (present study) and *C. etunicatum* (our previous work) is not possible due to data availability. Future studies should directly compare multiple AMF species using an integrated metabolomic pipeline to determine whether the extensive lipid reprogramming observed here is conserved or strain-specific. Building on these findings, future work should dissect the molecular dialog among AMF, root metabolites, and endophytic bacteria to clarify whether and how these interactions jointly contribute to enhanced potassium uptake and carbohydrate accumulation in sweet potato.

## 5. Conclusions

*Glomus intraradices* inoculation was associated with promoted sweet potato seedling growth and increased potassium uptake and the accumulation of starch and water-soluble carbohydrates, without significantly altering plant nitrogen or phosphorus uptake. These responses were accompanied by reduced rhizosphere electrical conductivity, increased organic matter content, extensive root metabolic reprogramming, and compartment-specific shifts in rhizosphere and endophytic bacterial communities. AMF inoculation was particularly associated with enhanced phenylpropanoid biosynthesis and lipid-related metabolism, increased rhizosphere bacterial diversity, and selective enrichment of endophytic taxa such as *Devosia* and *Niastella*. Together, these findings suggest that AMF-associated growth promotion in sweet potato is linked to coordinated changes in rhizosphere conditions, root metabolism, and bacterial community assembly, although the underlying causal relationships require further experimental validation.

## Data Availability

The raw sequence data of microbial community sequencing have been deposited in the NCBI Sequence Read Archive database under BioProject ID PRJNA1454692.

## Supplementary Materials

The following supporting information can be downloaded at: https://www.mdpi.com/article/10.3390/jof12060393/s1, Figure S1: Differences in sweet potato root metabolites under CK and AMF treatments; Figure S2: AMF differentially expressed metabolites identified as phenylpropanoids and polyketides; Table S1: Hormone-associated metabolites regulated by AMF in root of sweet potato; Figure S3: Relative abundance of bacterial composition at phyla and genera levels for different treatments; Table S2: Effects of root and AMF on relative abundance of bacterial composition at phyla and genera levels for different treatments; Table S3: Differentially abundant genera identified by LEfSe with logarithmic LDA > 2.5 between endophytic and rhizosphere bacterial communities; Table S4: Differentially abundant genera identified by LEfSe with logarithmic LDA > 2.5 between AMF and CK treatments in endophytic bacterial communities; Table S5: Differentially abundant genera identified by LEfSe with logarithmic LDA > 2.5 between AMF and CK treatments in rhizosphere bacterial communities; Table S6: Selective enrichment of endophytic bacteria from the rhizosphere in CK and AMF treatment; Table S7: Effects of root and AMF on bacterial functions.

## Abbreviations

The following abbreviations are used in this manuscript:

AMF	Arbuscular mycorrhizal fungi
PLS-DA	Partial least squares-discriminant analysis
FC	Food change
OTUs	Operational taxonomic units
PCA	Principal component analysis
LEfSe	Linear discriminant analysis effect size
LDA	Linear discriminant analysis
NMDS	Non-metric multidimensional scaling
